# Alkali Content as a Tool for Tailoring ZSM-48 Physicochemical Properties: From Crystallization Kinetics to Catalytic Performance in n-Hexadecane Hydroisomerization

**DOI:** 10.3390/molecules31162900

**Published:** 2026-08-20

**Authors:** Dmitry V. Serebrennikov, Arthur I. Malunov, Arthur R. Zabirov, Nadezhda A. Filippova, Alexandra D. Zimina, Alfira N. Khazipova, Ekaterina S. Mescheryakova, Rufina A. Zilberg, Marat R. Agliullin

**Affiliations:** 1 Institute of Petrochemistry and Catalysis, Ufa Federal Research Centre of the Russian Academy of Sciences, 450075 Ufa, Russia; d25c25@yandex.ru (D.V.S.); malunovv@mail.ru (A.I.M.); best535@inbox.ru (A.R.Z.); nelya-zimina@mail.ru (A.D.Z.); khazipova@gmail.com (A.N.K.); katusha2974@gmail.com (E.S.M.); maratradikovich@mail.ru (M.R.A.); 2 Department of Analytical Chemistry, Ufa University of Science and Technology, 450076 Ufa, Russia; zilbergra@yandex.ru

**Keywords:** RFE, ZSM-48 zeolite, reaction kinetics, n-hexadecane hydroisomerization

## Abstract

The morphology and pore structure of ZSM-48 zeolite are critical parameters determining the catalytic performance of bifunctional catalysts in the hydroisomerization of long-chain n-paraffins. This study investigates the effects of the Na_2_O/SiO_2_ molar ratio (0.02–0.12) in the synthesis gel and hydrothermal treatment duration (48–72 h) on the crystallization kinetics, phase purity, and physicochemical properties of ZSM-48. Low alkalinity (Na_2_O/SiO_2_ = 0.04–0.06) and shorter synthesis times (48 h) promote the formation of small aggregates composed of short needle-like crystals with enhanced intercrystalline mesoporosity. Conversely, increasing the alkalinity and crystallization duration accelerates crystal growth, resulting in dense pseudo-spherical aggregates (up to 4–7 μm in size) with restricted external surface area and increased diffusion limitations. Catalytic testing of Pt/ZSM-48 (0.5 wt.% Pt) in n-hexadecane hydroisomerization demonstrates that crystal morphology, size, and porosity significantly influence process selectivity. The catalyst based on nanosized ZSM-48 (Pt/Z48-06-2) effectively mitigates diffusion resistance, yielding a maximum isomer yield of 73% at 82% selectivity. In contrast, larger, densely packed aggregates with high but poorly accessible acidity intensify secondary hydrocracking reactions, reducing a maximum isomer yield to 46%. These results highlight the ability to tune the catalytic properties of ZSM-48 through careful control over gel alkalinity and crystallization kinetics.

## 1. Introduction

The production of high-quality, low-pour-point diesel fuels, including those derived from bio-based feedstocks, as well as advanced API Group III base oils, is a critical challenge in modern petroleum refining [1]. Stringent operational and environmental regulations, combined with the necessity for reliable machinery performance in cold-climate regions, impose rigorous requirements on the low-temperature properties of petroleum products. Higher n-paraffins (C_16+_) are key feedstock components that determine the quality of the final products. On one hand, they exhibit favorable physicochemical properties, providing high cetane numbers in diesel fractions and a high viscosity index in base oils. On the other hand, due to their linear molecular structure, n-paraffins undergo crystallization, which negatively affects the low-temperature performance of fuels and lubricants [2]. Traditional approaches to addressing this issue, such as solvent dewaxing or selective hydrocracking, involve the physical removal or degradation of n-paraffins, which inevitably leads to a significant decrease in the yield of valuable liquid products and a reduction in the viscosity index. In contrast, catalytic hydroisomerization (isodewaxing) is a highly efficient and resource-conservative industrial process that enables the conversion of linear molecules into branched isomers without substantial loss of molecular weight [2,3,4].

The crystal size, morphology, and pore structure of the zeolite component within a bifunctional catalyst play a fundamental role in this process. Zeolites with a one-dimensional channel system and a pore size of approximately 5 Å (formed by 10-membered oxygen rings) are widely employed in the hydroisomerization of higher paraffins. These include ZSM-23 (MTT topology), ZSM-22 (TON), and ZSM-48 (RFE, formerly MRE) [5,6]. Among these, ZSM-48 has attracted particular interest; its unique linear channel geometry and moderate acidity provide an optimal balance between reagent diffusion and the spatial constraints required for selective isomerization while minimizing undesirable secondary hydrocracking [7,8].

However, the efficiency of catalysts based on microporous zeolites, including ZSM-48, is often constrained by significant diffusion limitations [5,9]. Slow mass transfer of reagents and bulky isomerized products within narrow micropores leads to reduced reaction rates and decreased selectivity toward target products resulting from an increased contribution of hydrocracking reactions.

One of the most promising approaches to overcoming diffusion limitations is the development of nanosized or hierarchical materials. In such systems, the presence of a secondary network of meso- or macropores significantly reduces the diffusion path length within micropores and facilitates the transport of molecules to active sites [10]. Modern literature describes several methods for regulating the pore structure and crystal size of ZSM-48. Techniques employing specific crystal growth modifiers that restrict zeolite growth along certain crystallographic directions are actively being developed [11,12]. A prominent role is occupied by strategies using bifunctional templates, such as the DGC method (gemini quaternary ammonium compounds), in which a single complex organic molecule acts simultaneously as a structure-directing agent (SDA) for micropores and a template for mesopore formation [13,14,15]. In addition to these methods, considerable attention is given to creating hierarchical porosity via post-treatment leaching with alkali, acid, or steam [16,17,18].

However, despite the high efficiency of these methods in generating well-developed hierarchical structures, their application is associated with significant drawbacks. The use of expensive modifiers, complex bifunctional SDAs, and multi-stage post-treatment leaching procedures renders these approaches difficult to scale and economically unfeasible for industrial production.

It is important to note that the topology and specific crystallization characteristics of ZSM-48 zeolite offer a more rational pathway for the construction of hierarchical systems, in particular, a nanosized structure with developed secondary porosity. Hexamethonium bromide is traditionally the most widely used and effective structure-directing agent (SDA) for the synthesis of ZSM-48 [6]. Unlike ZSM-23 and ZSM-22, which typically crystallize as long, dense needles with high aspect ratios [19,20,21,22], the use of hexamethonium bromide facilitates the formation of ZSM-48 as shortened, needle-like nanocrystals capable of self-assembling into sponge-like aggregates [19,23,24]. In other words, ZSM-48 synthesized via standard procedures inherently possesses a predisposition for the formation of a nanosized structure with developed secondary porosity.

The scientific literature also describes ZSM-48 synthesized as long needles, large rods, or massive spherical aggregates [17,25,26,27,28,29]. The enlargement of crystals and their dense aggregation negate the diffusion benefits of the RFE framework, sharply increasing transport resistance. This significant structural diversity is precisely what accounts for the contradictory reports in the literature regarding the catalytic performance of ZSM-48-based systems. Consequently, the development of synthesis methods for nanosized and hierarchical ZSM-48 that avoid the use of expensive crystal growth modifiers and additional surfactants is a highly relevant research objective.

In this context, understanding the correlation between crystallization parameters and the resulting morphology is a critical task for the rational synthesis of accessible and efficient isodewaxing catalysts. In this work, we demonstrate for the first time how simple control over the alkalinity (Na_2_O/SiO_2_ ratio) and crystallization kinetics allows for fine-tuning of the physicochemical properties of ZSM-48 zeolite, including its crystallinity, morphology, pore structure, and acid characteristics. The established relationships enable the targeted formation of both optimal mesoporous nanoaggregates and large, dense spherulites, and clearly demonstrate how these structural differences ultimately determine the catalytic performance of Pt/ZSM-48 in the hydroisomerization of n-hexadecane.

## 2. Results

**Figure 1:**
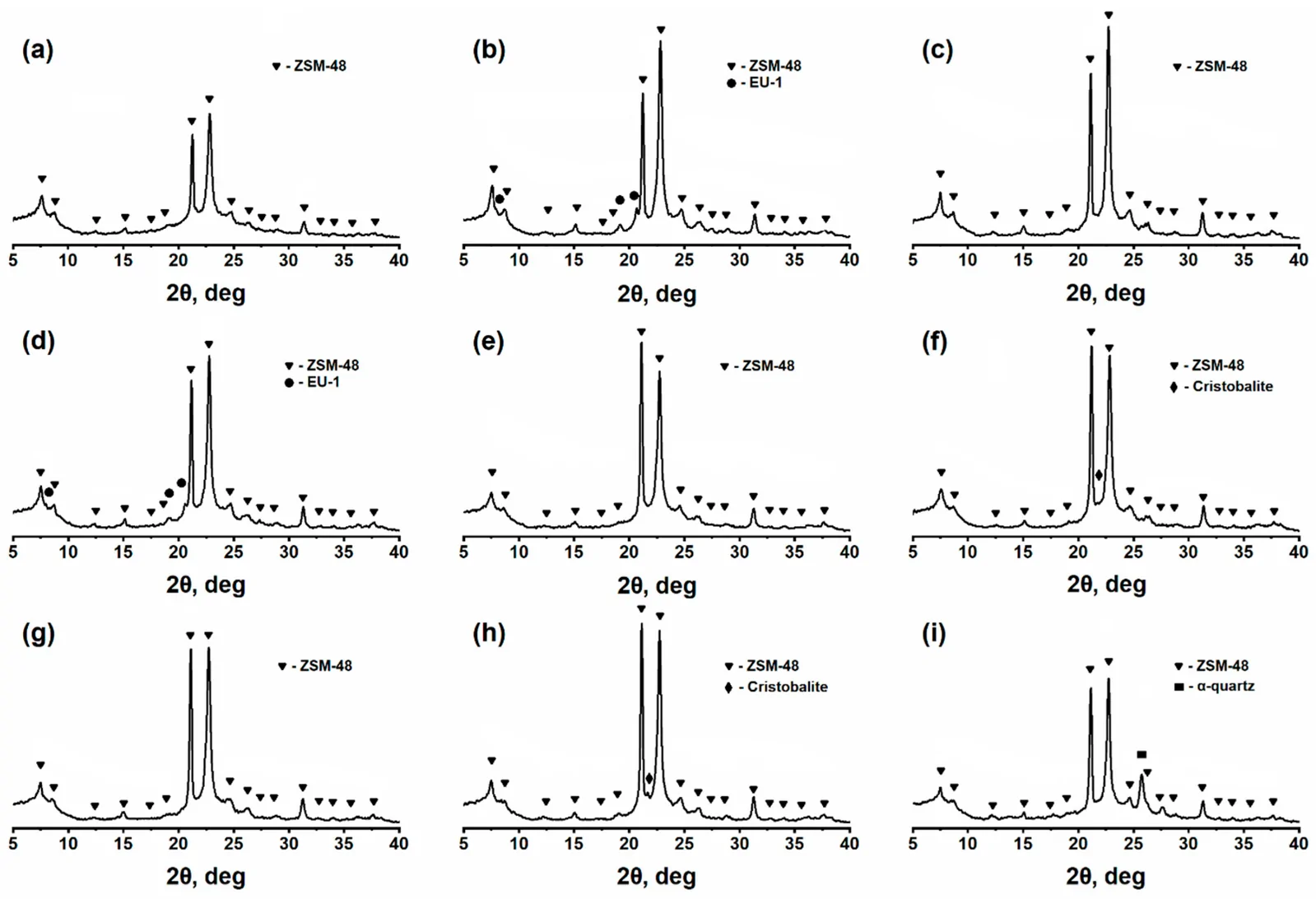
X-ray diffraction patterns of the crystallization products: (**a**) Z48-04-2, (**b**) Z48-04-3, (**c**) Z48-06-2, (**d**) Z48-06-3, (**e**) Z48-08-2, (**f**) Z48-08-3, (**g**) Z48-10-2, (**h**) Z48-10-3, (**i**) Z48-12-2.

The crystallization kinetics of ZSM-48 zeolite were investigated at a constant temperature of 160 °C and a fixed SiO_2_/Al_2_O_3_ molar ratio of 140 in the parent gel. Variations in synthesis conditions were restricted to the alkalinity of the medium (Na_2_O/SiO_2_ molar ratio, hereinafter denoted as x), ranging from 0.02 to 0.12, and the duration of crystallization. At the minimum value of x = 0.02, the material remained entirely amorphous even after 72 h of crystallization, indicating that the hydroxide ion concentration was insufficient to initiate the nucleation of the RFE topology (Table 1, Figure 1). Increasing the alkali content to x = 0.04 significantly accelerated the process: after 48 h, a crystallinity of 76% was achieved with the formation of phase-pure ZSM-48; however, extending the synthesis to 72 h resulted in the appearance of trace amounts of the competing EU-1 zeolite phase.

The optimal crystallization window is observed in the range of x = 0.06–0.10, where high crystallinity (89–90%) and a phase-pure ZSM-48 are achieved within 48 h. However, extending the synthesis time to 72 h within this composition range again leads to the formation of impurities: traces of EU-1 are detected at x = 0.06, whereas non-porous cristobalite appears at x = 0.08–0.10. A further increase in alkalinity to x = 0.12 results in a mixture of ZSM-48 and α-quartz.

Elemental analysis of the crystallization products revealed a distinct dependence of aluminum incorporation into the zeolite framework on both alkalinity and synthesis duration. It was established that for a 72 h crystallization, increasing the Na_2_O/SiO_2_ ratio in the gel from 0.04 to 0.10 leads to a monotonic decrease in the SiO_2_/Al_2_O_3_ molar ratio in the zeolite from 135 to 116. Conversely, reducing the synthesis time to 48 h promotes the formation of more siliceous products. For instance, at x = 0.06, the SiO_2_/Al_2_O_3_ ratio in the solid phase after 48 h is 128 (compared to 119 after 72 h). This trend indicates that ZSM-48 silicate nuclei are predominantly formed and grown during the initial stages. An increase in synthesis duration and alkali concentration facilitates the dissolution of the aluminum source and its subsequent incorporation into the framework, or stimulates secondary crystallization of a material with higher aluminum content.

In addition to chemical composition, varying the alkalinity significantly influences the structural perfection of the ZSM-48 framework, which can be interpreted through changes in the relative intensities of the diffraction peaks. With an increasing Na_2_O/SiO_2_ ratio, the intensity of the reflection at 2θ ≈ 7.61° (110) decreases progressively, whereas the intensity of the reflection at 2θ ≈ 21.25° (002) increases. Since zeolites of the RFE family are prone to forming one-dimensional stacking faults along the c-axis, which coincides with the direction of the 10-membered ring channels [8,30], these reflections exhibit varying sensitivities to the coherence of the structural blocks. The (110) reflection corresponds to long-range order in the ab-plane and is weakly dependent on longitudinal shifts, whereas the intensity of the (002) reflection is directly determined by the regularity of layer stacking along the c-axis. Consequently, the observed increase in the I(002)/I(110) ratio with increasing alkalinity can be considered a reliable semi-quantitative indicator suggesting improved structural coherence along the channel direction, which is consistent with a reduction in the fraction of “shifted framework” blocks. Thus, a high alkali content not only accelerates crystallization kinetics but also likely promotes the minimization of stacking faults, leading to a more ordered framework, whereas in low-alkalinity media, kinetic limitations lead to the fixation of disordered structural blocks.

**Figure 2:**
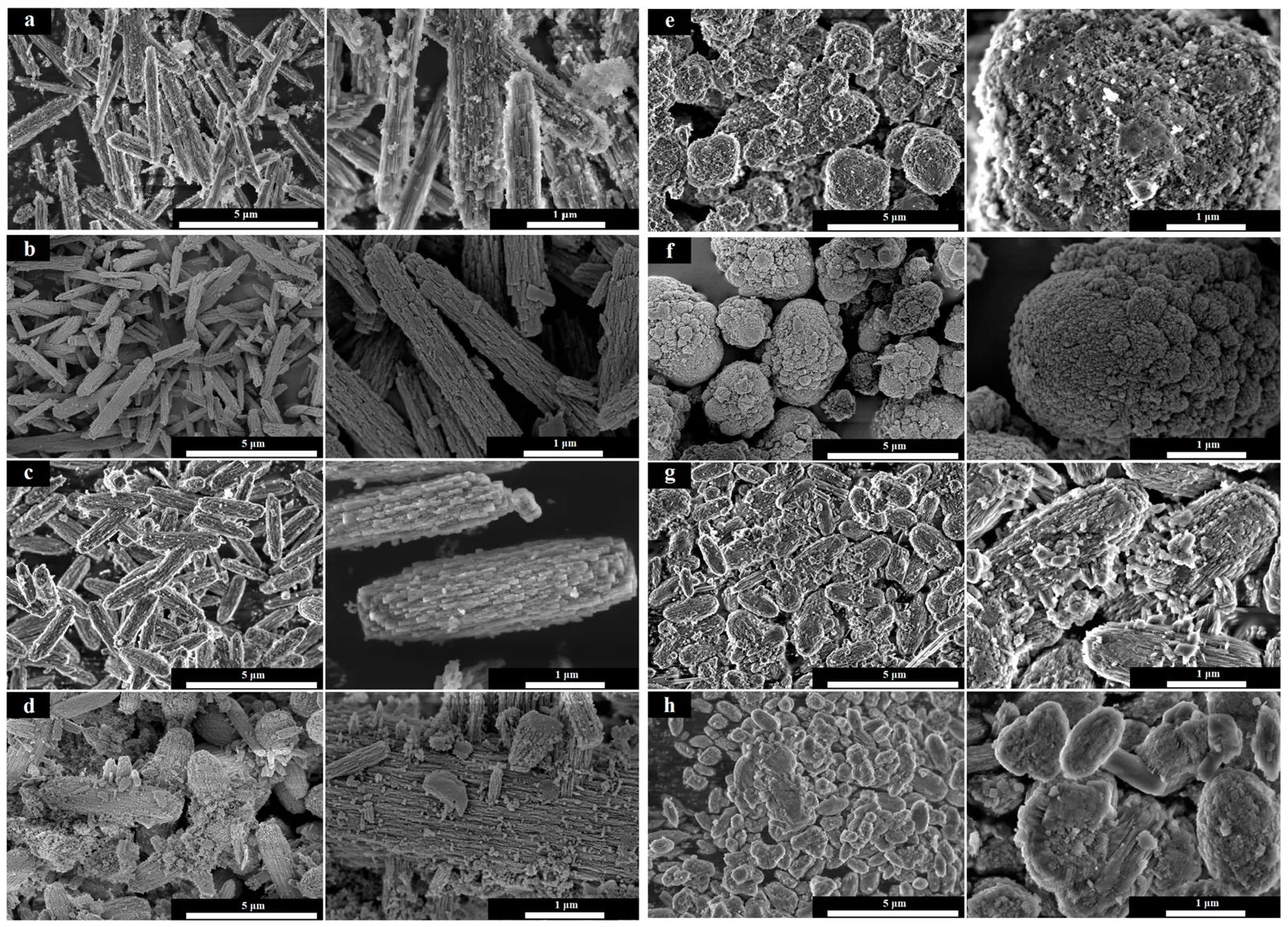
SEM images of ZSM-48 zeolite obtained at different crystallization times and Na_2_O/SiO_2_ molar ratios in the parent gel: (**a**) Z48-04-2; (**b**) Z48-04-3; (**c**) Z48-06-2; (**d**) Z48-06-3; (**e**) Z48-08-2; (**f**) Z48-08-3; (**g**) Z48-10-2; (**h**) Z48-10-3.

**Figure 3:**
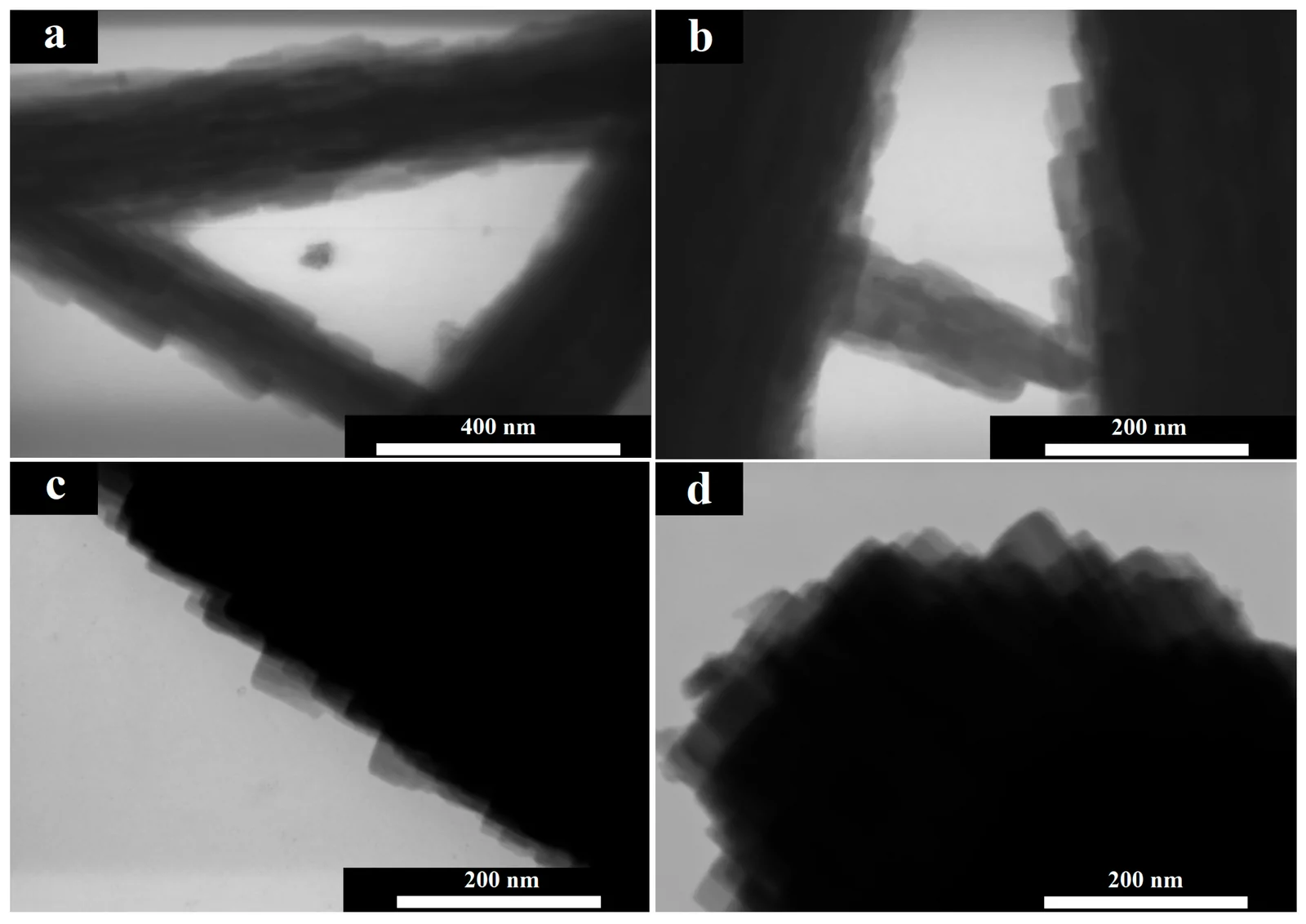
STEM images of ZSM-48 zeolite obtained at different crystallization times and Na_2_O/SiO_2_ molar ratios in the parent gel: (**a**) Z48-04-3; (**b**) Z48-06-2; (**c**) Z48-06-3; (**d**) Z48-10-3.

Morphological characterization by scanning electron microscopy (SEM, Figure 2) revealed a dependence of crystal size and aggregation degree on the synthesis parameters. At minimum alkalinity (x = 0.04, 72 h), elongated needle-like aggregates typical of ZSM-48 are formed (diameter ~500 nm, length 3–5 μm), composed of densely packed small crystallites. High-resolution scanning transmission electron microscopy (STEM, Figure 3) analysis revealed that these crystallites have dimensions of approximately 20–40 nm in diameter, confirming the nanoscale nature of the primary building units. Interestingly, when the crystallization time was reduced to 48 h, the individual needle-like crystals became more clearly distinguishable within the aggregates, suggesting less extensive intergrowth at earlier stages of crystallization. However, this sample also exhibited a visible amorphous layer coating the surface of the aggregates, which is consistent with the incomplete crystallization (76%) observed by XRD at this shorter synthesis time. This amorphous material likely represents residual unreacted gel or poorly ordered aluminosilicate species that have not yet been incorporated into the crystalline framework.

At moderate alkalinity and reduced synthesis time (sample Z48-06-2), the material is formed predominantly from nanosized crystals smaller than 100 nm (Figure 3) with a low aspect ratio, which assemble into small, loose aggregates (diameter 300–700 nm, length ~2 μm). Such a unique nanosized morphology is typical of the early stages of crystallization and offers the potential to significantly improve the diffusion of bulky molecules by substantially shortening the length of the microporous channels. Extending the hydrothermal treatment of this composition to 72 h (Z48-06-3) induces crystal growth and their coalescence into large conglomerates up to 2 × 7 μm.

The most significant morphological changes occur as the alkalinity increases to x = 0.08. The classical needle-like morphology transforms into “cauliflower-like” spherical formations with a diameter of 4–5 μm (after 48 h, Z48-08-2), which grow to 4–7 μm after 72 h (Z48-08-3). Presumably this transition to an isometric (spherulitic) shape is driven by the specific adsorption of sodium ions on certain crystallographic faces, which suppresses their linear growth. Notably, these spherulites exhibit the lowest degree of structural disorder (maximum I(002)/I(110) ratio). At shorter crystallization times for this composition (Z48-08-2), the surfaces of the aggregates are decorated with numerous ultrafine amorphous particles adjacent to larger, irregularly shaped objects that are likely early-stage crystalline nuclei. However, it is crucial to note that despite this surface heterogeneity, XRD analysis confirms that the bulk of this material is already highly crystalline ZSM-48. This indicates that the observed amorphous surface features represent only a minor, transient fraction, while the core framework has already achieved a high degree of structural ordering.

At maximum alkalinity (x = 0.10), the system becomes heterogeneous, demonstrating a polymodal crystal distribution (clusters of long needles and large short fragments). This pronounced heterogeneity suggests that at such high alkalinity, the system experiences competing nucleation and growth regimes, preventing the formation of a uniform morphological product.

**Figure 4:**
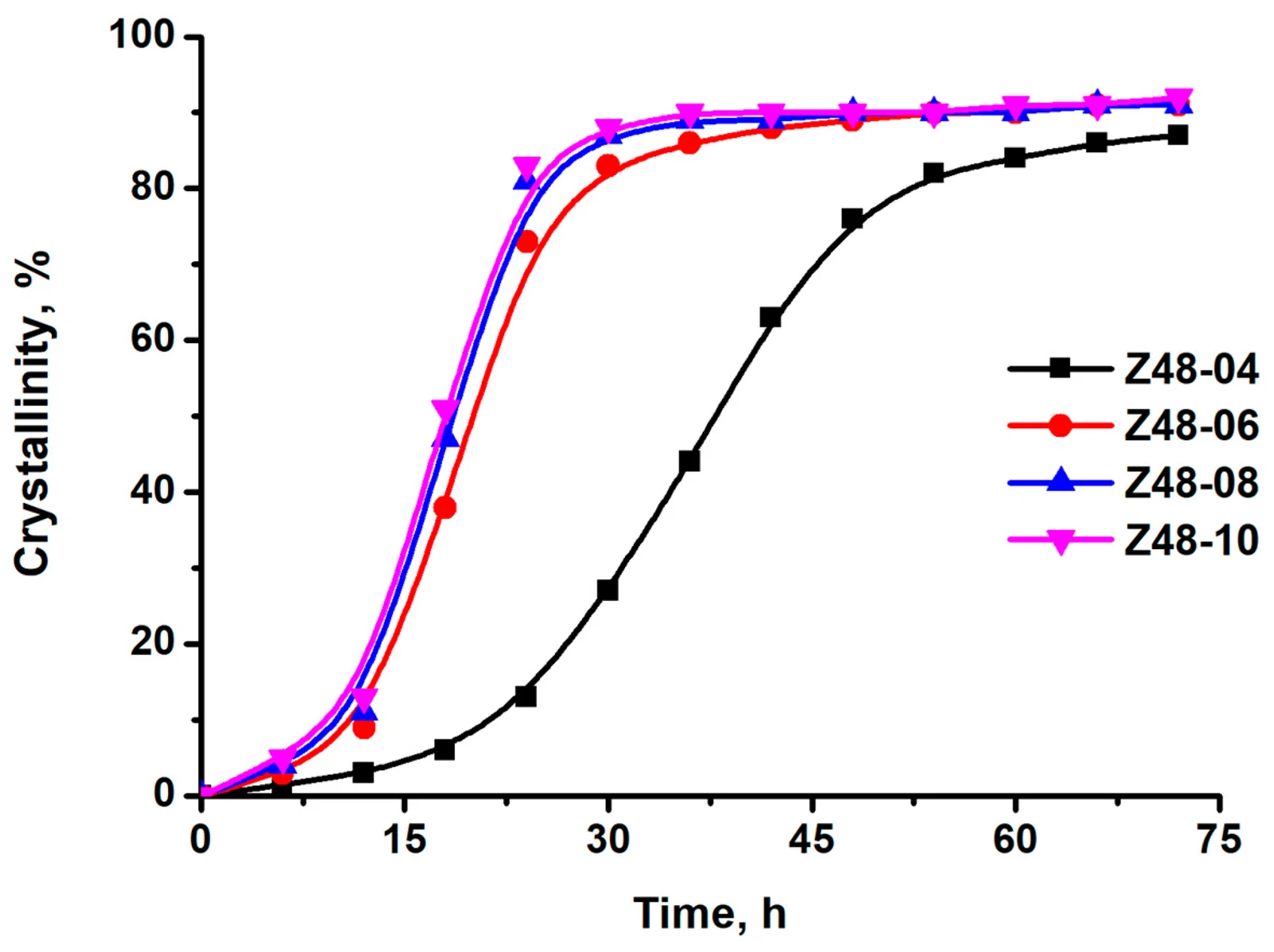
Crystallization kinetic curves at different Na_2_O/SiO_2_ molar ratios (0.04–0.10) in the parent gel.

The constructed crystallization kinetic curves (Figure 4) exhibit a classical S-shaped profile and are in good agreement with the SEM data. At the minimum investigated value of Na_2_O/SiO_2_ = 0.04, the induction period is approximately 24 h. The amorphous nature of the material at the end of this induction period is confirmed by SEM imaging (Figure S1a,b), which reveals no discernible crystalline features after 24 h of hydrothermal treatment. This prolonged initial stage is due to the low concentration of hydroxide ions, which limits the rate of amorphous silica depolymerization. This retarded kinetics explains the morphological features: due to the deficit of building units, the crystals do not have sufficient time to grow to larger sizes.

Increasing the Na_2_O/SiO_2_ ratio to 0.06, 0.08, and 0.10 reduces the induction period to ~12 h in all three cases. At this 12 h mark, the material remains predominantly amorphous (Figure S1c,e). However, by 24 h, well-defined aggregates of partially crystallized ZSM-48 have already formed (Figure S1d,f). Crucially, this 24 h time point corresponds to the completion of the rapid growth stage on the kinetic curves, indicating that the primary crystallization event occurs within this narrow 12-to-24 h window. However, differences are observed during the accelerated growth stage. At x = 0.06, the growth rate remains more moderate, which is reflected in the morphology: short aggregates form within 48 h, and by 72 h, as the kinetic curve reaches a plateau, they coarsen and fuse into larger conglomerates.

**Figure 5:**
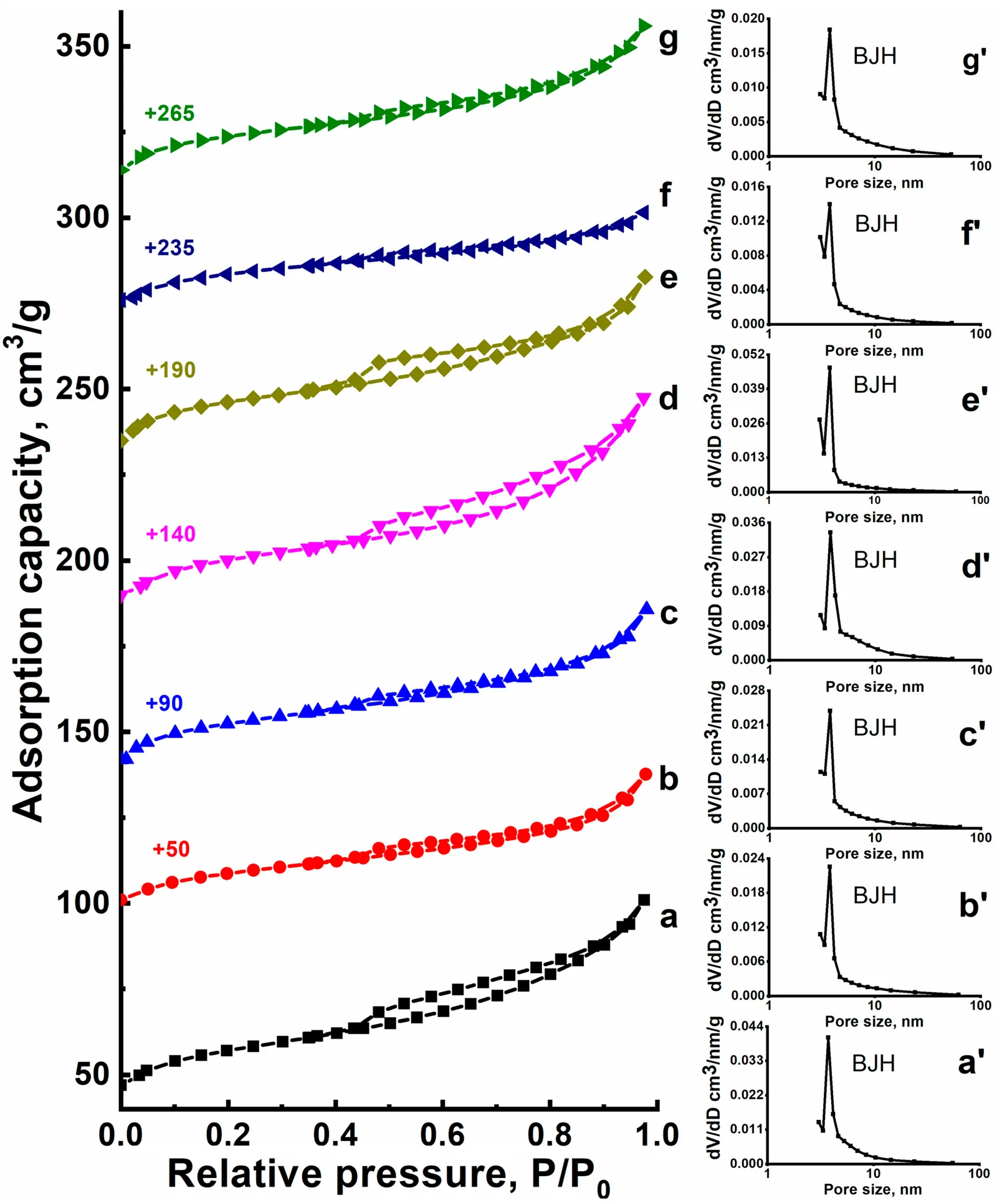
N_2_ adsorption–desorption isotherms and BJH pore size distributions: (**a**,**a’**) HZ48-06-2; (**b**,**b’**) HZ48-08-2; (**c**,**c’**) HZ48-10-2; (**d**,**d’**) HZ48-04-3; (**e**,**e’**) HZ48-06-3; (**f**,**f’**) HZ48-08-3; (**g**,**g’**) HZ48-10-3.

Low-temperature nitrogen adsorption–desorption analysis (Figure 5, Table 2) confirmed the presence of distinct textural differences. The samples Z48-04-3 and Z48-06-2 obtained at low alkali content (x = 0.04–0.06) exhibit Type IV isotherms with a well-defined hysteresis loop. These are characterized by maximum values of external surface area (S_EXT_ = 58 m^2^/g for Z48-06-2) and the highest mesopore volume (0.09 cm^3^/g), attributed to the loose packing of nanosized crystals. The presence of mesopores in the 4–15 nm range is clearly visible on the BJH pore size distribution curves. While the sharp peak at 3.8 nm (ascribed to the cavitation phenomenon during desorption) partially overlays this area, a well-defined shoulder corresponding to the intercrystalline porosity of the ZSM-48 nanocrystals is observed. In contrast, the large aggregates of the high-alkali series (Z48-08-2, Z48-10-2) are characterized by Type IV isotherms, but with significantly less pronounced hysteresis. This confirms the denser packing of crystals with a minimal contribution from mesoporosity (0.03 cm^3^/g for Z48-08-3).

**Figure 6:**
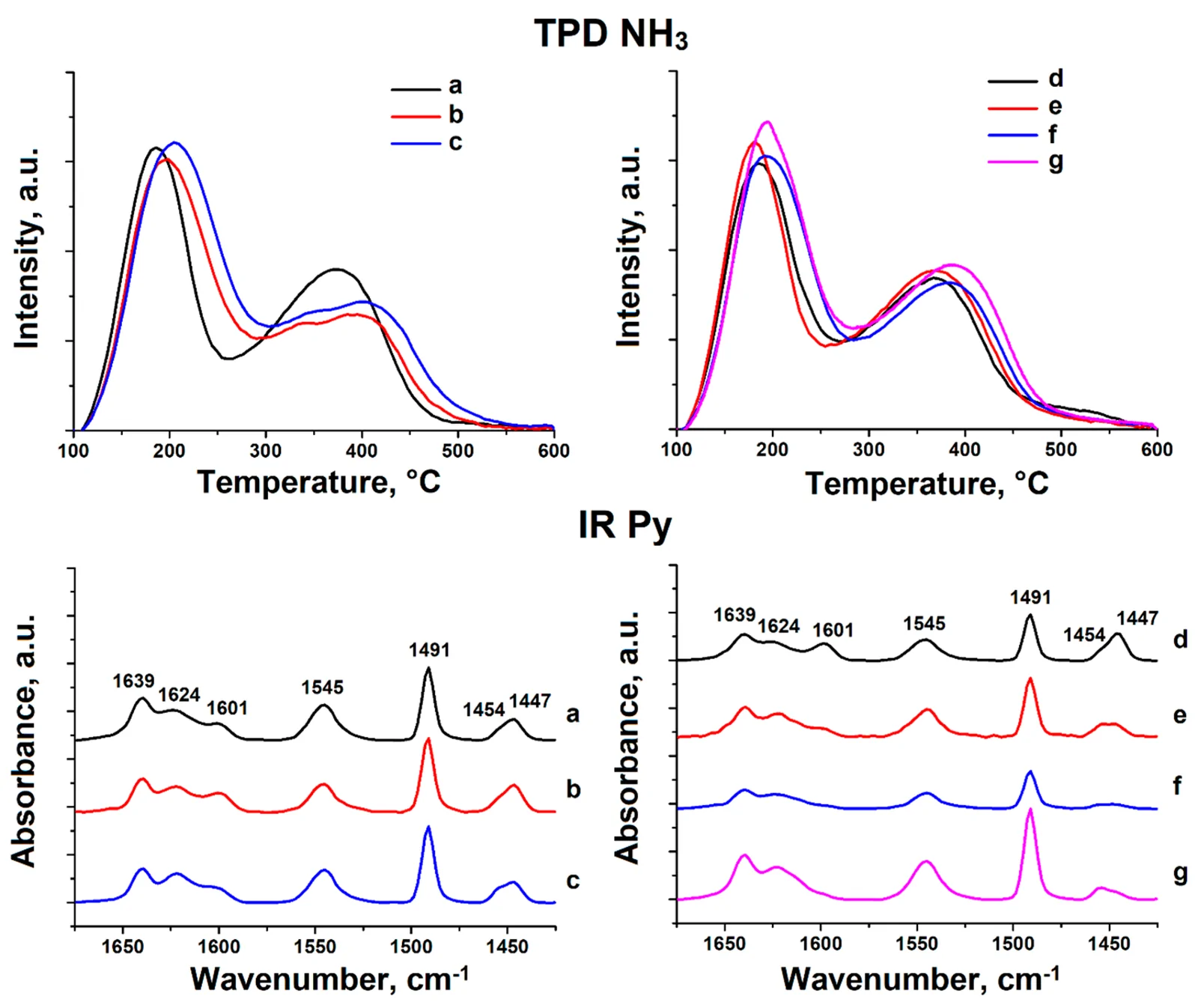
Acidity of ZSM-48 zeolite samples according to NH_3_ temperature-programmed desorption (NH_3_-TPD) and FTIR spectroscopy of adsorbed pyridine (IR Py): (**a**) HZ48-06-2; (**b**) HZ48-08-2; (**c**) HZ48-10-2; (**d**) HZ48-04-3; (**e**) HZ48-06-3; (**f**) HZ48-08-3; (**g**) HZ48-10-3.

The analysis of the acidic properties of ZSM-48 in H-form by NH_3_-TPD and FTIR spectroscopy of adsorbed pyridine revealed a complex dependence on the physicochemical properties of the synthesized materials. The NH_3_-TPD curves (Figure 6) demonstrate three characteristic desorption regions: 100–250 °C (weak acid sites), 250–350 °C (medium-strength sites), and 350–600 °C (strong acid sites). The total concentration of acid sites (Table 3) consistently increases as the Si/Al molar ratio in the zeolite decreases (Table 1). For instance, at a crystallization time of 48 h, the total concentration of sites increases from 195 to 223 μmol/g as Na_2_O/SiO_2_ increases from 0.06 to 0.10, while the concentration of strong sites increases from 68 to 72 μmol/g. A similar trend is observed for samples after 72 h of crystallization: an increase from 198 to 229 μmol/g (total) and from 50 to 84 μmol/g (strong sites) within the same alkalinity range. An exception was observed for samples obtained at Na_2_O/SiO_2_ = 0.08, which, as shown previously, are characterized by the formation of the largest and most dense spherical aggregates. For these samples, the concentration of strong acid sites is lower, amounting to only 59 μmol/g (HZ48-08-2) and 47 μmol/g (HZ48-08-3).

In the FTIR spectra of all samples, Brønsted acid sites (BAS) are clearly identified by characteristic bands at 1639 and 1545 cm^−1^. Lewis acid sites (LAS) are represented by two types: the first (bands at 1622 and 1454 cm^−1^) corresponds to strong sites associated with aluminum in the zeolite framework, while the second (bands at 1601–1604 and 1457 cm^−1^) refers to weak LAS, which are most likely due to the presence of extra-framework aluminum [31]. Notably, in all samples studied, the majority of LAS are desorbed at 250 °C, which confirms the predominance of weak sites and indicates that the main source of the observed LAS is extra-framework aluminum.

Quantitative analysis of the FTIR spectra showed that increasing the alkali content and crystallization time leads to an increase in the concentration of BAS, while the concentration of LAS decreases. For example, at minimum alkalinity (Na_2_O/SiO_2_ = 0.04), the BAS concentration is 78 μmol/g, and the LAS concentration is 49 μmol/g. As the Na_2_O/SiO_2_ ratio increases to 0.10, the BAS content rises to 95 μmol/g (HZ48-10-2) and 120 μmol/g (HZ48-10-3), whereas the LAS concentration drops to 36 and 19 μmol/g, respectively. In addition to the chemical factor, a pronounced correlation is observed between the acid site concentrations and the size and density of the aggregates. Pyridine, possessing a larger kinetic diameter and molar volume compared to ammonia, is sensitive to textural constraints. In the sample with small aggregates and short crystals (Na_2_O/SiO_2_ = 0.06, 48 h), the concentrations are 93 μmol/g (BAS) and 37 μmol/g (LAS). In contrast, for the sample with larger aggregates (HZ48-08-3), a sharp drop to 59 μmol/g (BAS) and 11 μmol/g (LAS) is recorded. The adsorption of pyridine can sterically block the 1D channels, hindering the diffusion of subsequent molecules to deeper-located acid sites. This diffusion limitation is amplified in samples with larger aggregates and reduced mesopore volume, leading to a more significant underestimation of acidity by the FTIR-Py method compared to NH_3_-TPD.Furthermore, the spatial distribution of aluminum atoms within the zeolite framework likely varies depending on synthesis conditions, which may additionally affect the accessibility of acid sites to pyridine. For instance, the exceptionally low pyridine-measured acidity in HZ48-08-3 may be attributed to aluminum atoms being preferentially located deeper within the crystalline framework, rendering them less accessible to the bulky pyridine probe. Conversely, HZ48-10-3 exhibits the highest pyridine-accessible acidity, suggesting that under these highly alkaline conditions, aluminum incorporation occurs preferentially in more accessible, near-surface positions of the crystals. It is likely that increasing the amount of alkali promotes better incorporation of aluminum into the framework of zeolite ZSM-48. In addition, it is likely that an increase in the amount of alkali contributes to a better incorporation of aluminum into the structure of zeolite ZSM-48, according to the content of weak LAS.

The catalytic properties of the synthesized samples were studied in the hydroisomerization of n-hexadecane using platinum-based catalysts. To deposit the platinum and conduct subsequent tests, samples with the most contrasting physicochemical characteristics were selected, which allowed for a clear demonstration of the influence of morphology, texture, and acid properties of the support on catalytic performance: Pt/Z48-06-2, Pt/Z48-04-3, Pt/Z48-06-3, Pt/Z48-08-3, and Pt/Z48-10-3. The active metal was deposited by incipient wetness impregnation using a chloroplatinic acid solution with a target loading of 0.5 wt.% Pt. According to hydrogen pulse chemisorption data on pre-reduced samples, the platinum particle size averaged 3–4 nm (Table 4) in all cases, indicating a similar dispersion of the metal phase and allowing the observed differences in catalytic properties to be attributed exclusively to the features of the acidic support—the ZSM-48 zeolite.

**Figure 7:**
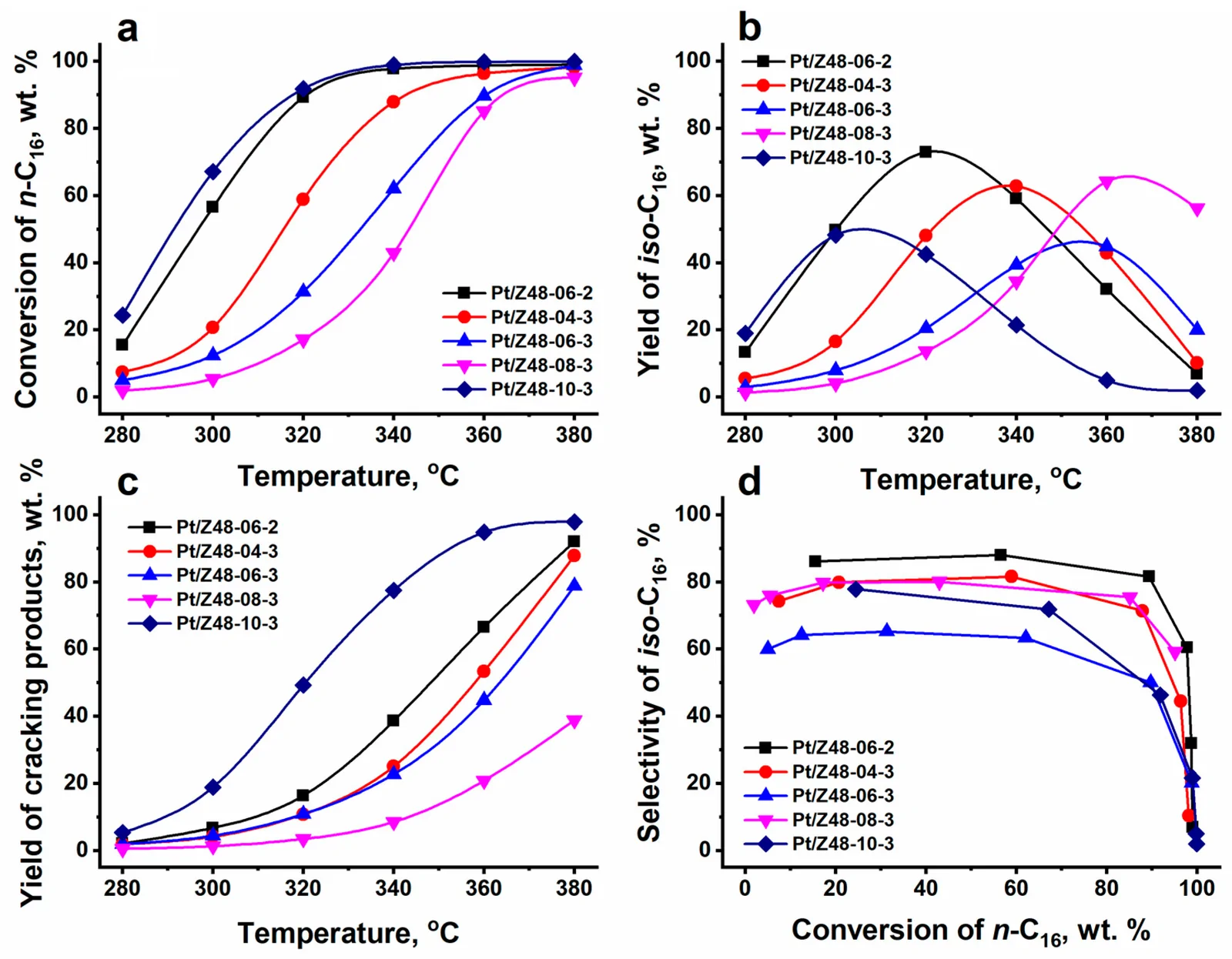
Hydroisomerization of n-hexadecane over Pt/ZSM-48 samples: (**a**) n-hexadecane conversion as a function of reaction temperature; (**b**) yield of C_16_ isomers as a function of reaction temperature; (**c**) yield of hydrocracking products as a function of reaction temperature; (**d**) selectivity to hexadecane isomers as a function of n-hexadecane conversion.

As the reaction temperature increases, the conversion of n-hexadecane increases monotonically for all catalysts (Figure 7). Since both isomerization and hydrocracking products accumulate in parallel as conversion increases, the dependence of isomer yield on conversion passes through a maximum, after which a sharp decrease in selectivity toward target products is observed due to the predominance of secondary reactions involving the cleavage of the hydrocarbon skeleton.

The highest activity in the hydroconversion of n-hexadecane was demonstrated by two samples: the nanosized Pt/Z48-06-2 and the most acidic Pt/Z48-10-3. However, their catalytic behavior differs fundamentally in terms of selectivity. The Pt/Z48-06-2 sample, characterized by small aggregates of short crystals, high external surface area, and developed intercrystalline mesoporosity, proved to be the most selective in the isomerization reaction: at 89% conversion, the selectivity to hexadecane isomers was 82%, and the maximum isomer yield reached 73%. In contrast, on Pt/Z48-10-3, which is distinguished by a high concentration of strong acid sites but formed from large aggregates, the isomer selectivity did not exceed 46% at the same conversion, and the maximum yield was only 42%. This low selectivity, despite high activity, is primarily attributed to its high concentration of strong acid sites (Table 3), which can be promote the formation of highly branched isomers prone to hydrocracking. This effect is exacerbated by diffusion limitations within its large aggregates. Furthermore, pyridine adsorption data suggests that the specific distribution of aluminum in this sample may render these strong sites particularly accessible, further accelerating cracking reactions.

The Pt/Z48-08-3 sample, characterized by the lowest concentration of acid sites due to its large, dense spherical aggregates, demonstrated the lowest activity in the hydroconversion of n-hexadecane. However, its moderate selectivity (a maximum isomer yield of 64%, significantly higher than the 45% on the coarse-intergrowth Pt/Z48-06-3) may also be linked to its lower overall acidity and the potentially restricted accessibility of its acid sites, as indicated by the pyridine adsorption results. This limited accessibility likely suppresses severe hydrocracking, preserving isomer selectivity at the expense of overall conversion.

The Pt/Z48-04-3 sample, characterized by small but still larger aggregates compared to Pt/Z48-06-2, demonstrated intermediate results. Its lower activity relative to the optimal Pt/Z48-06-2 catalyst can be directly attributed to its lower concentration of accessible acid sites. Nevertheless, it exceeds Pt/Z48-06-3 and Pt/Z48-08-3 in activity, achieving a maximum yield of hexadecane isomers of 63%.

## 3. Materials and Methods

### 3.1. Synthesis of ZSM-48

The ZSM-48 zeolite was synthesized from a reaction gel with the following molar composition: xNa_2_O·1.0SiO_2_·0.007Al_2_O_3_·0.02HMBr·25H_2_O, where x ranged from 0.02 to 0.12. The reagents used for preparation included sodium hydroxide (NaOH, Reakhim, Moscow, Russia), silica sol (30% SiO_2_, Nanosil-30, Prom-Steklo, Yekaterinburg, Russia) as the silicon source, sodium aluminate (Reakhim, Moscow, Russia) as the aluminum source, and hexamethonium bromide (HMBr, Macklin, Shanghai, China) as the structure-directing agent (template). To prepare the reaction gel, sodium aluminate was first dissolved in a NaOH solution under vigorous stirring. The calculated amount of template was added to the resulting solution. Then, the silica sol was added slowly, dropwise, to the mixture. After mixing all components, the resulting gel was transferred to a steel autoclave, and hydrothermal crystallization was carried out at 160 °C for 6–72 h. The obtained solid product was separated by centrifugation, thoroughly washed with distilled water until neutral pH was reached, and dried at 110 °C. Hereinafter, the samples are designated as Z48-y-z, where y is Na_2_O/SiO_2_·100, and z denotes 2 days (48 h) or 3 days (72 h) of crystallization.

The zeolite samples were converted into their protonated (H-form) via a cation exchange procedure, wherein Na^+^ ions were replaced by NH_4_^+^ ions. The exchange was carried out in a 0.5 M aqueous solution of ammonium nitrate (NH_4_NO_3_, 98%, Reakhim, Moscow, Russia) with a liquid/solid ratio of 10 at 70 °C for 2 h under continuous stirring. The degree of exchange of Na^+^ ions for NH_4_^+^ ions of 0.99 was achieved for all samples. The resulting suspension was subsequently dried at 110 °C and calcined in an air atmosphere at 550 °C for 4 h. The final protonated materials were designated with an “H” index, i.e., HZ48-y-z.

### 3.2. Material Analysis Methods

The elemental analysis of the crystallized zeolites, and the platinum-containing catalysts was carried out via X-ray fluorescence (XRF) spectroscopy. A Shimadzu EDX-7000P system (Shimadzu Corporation, Duisburg, Germany) was used, with quantification based on the fundamental parameters method.

Powder X-ray diffraction (XRD) patterns for the as-synthesized (uncalcined) ZSM-48 samples were acquired using a Shimadzu XRD-7000 diffractometer (Shimadzu Corporation, Kyoto, Japan) with CuKα radiation. The scans were recorded from 5° to 40° (2θ) at a scanning rate of 1°/min. The PDF-2 database (version 2.2201) was utilized for phase identification. The degree of crystallinity was determined by calculating the ratio of the integrated intensity of the crystalline peaks to the total area under the curve (including the amorphous halo) in the 2θ range of 20–30°. This was performed using Shimadzu XRD Crystallinity software (version 7.04). To account for the uncertainty in baseline fitting in the presence of prominent Bragg reflections, the estimated error of the crystallinity determination is ±2%.

To evaluate the morphology and determine the crystal sizes of the ZSM-48 specimens, field-emission scanning electron microscopy (FE-SEM) was employed. The analyses were conducted on a Hitachi Regulus SU 8220 system (Hitachi High-Tech Corporation, Tokyo, Japan). Secondary electron signals were utilized to capture the images, operating the instrument at an accelerating voltage of 5 kV. It was also used in scanning transmission microscopy (STEM) mode. The accelerating voltage in this case was 30 kV.

Nitrogen adsorption–desorption isotherms at −196 °C were recorded on an Altamira Instruments QUICK-200 analyzer (Altamira Instruments Co., Beijing, China) to evaluate the textural properties, including specific surface area and micro/mesopore volumes. The specific surface area was calculated via the multi-point Brunauer–Emmett–Teller (BET) method. The t-plot method was applied to determine the volume and specific surface area of micropore. The specific external surface area was calculated as the difference between the total specific surface area according to BET and the specific surface area of micropores. The pore size distribution was derived from the desorption branch of the isotherm using the Barrett–Joyner–Halenda (BJH) model.

The total acidity and acid strength distribution were investigated by ammonia temperature-programmed desorption (NH_3_-TPD) on an Altamira AMI-400TPx apparatus (Altamira Instruments Co., Beijing, China). Before the analysis, the sample was calcined at 550 °C for 4 h. The system was then cooled to 100 °C, and the sample was saturated with a 10 vol% NH_3_/He gas mixture for 30 min. Physisorbed ammonia was removed by purging with helium at 100 °C. The desorption of ammonia was monitored with a thermal conductivity detector from 100 to 600 °C at a heating rate of 10 °C/min.

Fourier-transform infrared spectroscopy of adsorbed pyridine (IR-Py) was used to identify the types of acid sites (Brønsted, BAS, and Lewis, LAS) and quantify their concentrations. The spectra were recorded on a Bruker Vertex-70V spectrometer (Bruker Optic GmbH, Ettlingen, Germany) in the 4000–400 cm^−1^ range with a resolution of 4 cm^−1^. The samples were pressed into tablets (10 mg/cm^2^) and pretreated by calcination under vacuum (450 °C, 1 h). Pyridine was adsorbed for 30 min, followed by stepwise desorption at 150, 250, and 350 °C. The concentrations of BAS and LAS were calculated by integrating the absorption bands at 1545 cm^−1^ and 1454 cm^−1^, respectively, using the corresponding molar extinction coefficients [32].

### 3.3. Preparation of Pt/ZSM-48 Catalysts

To prepare the 0.5 wt% Pt/ZSM-48 catalysts, the incipient wetness impregnation procedure was employed. The ZSM-48 zeolite support was first calcined in air at 550 °C for 4 h. An aqueous solution of H_2_PtCl_6_·6H_2_O (Acros Organics, Morris Plains, NJ, USA) with a calculated concentration was then used to impregnate the support. The resulting wet samples were dried at 110 °C for 24 h, followed by calcination in an air flow at 550 °C for 5 h.

Hydrogen pulse chemisorption was carried out on an Altamira AMI-400TPx analyzer to determine the Pt dispersion and mean particle size. The pre-treatment protocol involved a reduction under a 50 mL/min H_2_ flow at 400 °C for 4 h. The chemisorption isotherms were recorded at 40 °C. A stoichiometric ratio of H/Pt = 1 was applied to calculate the metal dispersion and particle dimensions.

### 3.4. Hydroisomerization of n-Hexadecane

Hydroisomerization experiments were conducted in a tubular stainless-steel fixed-bed reactor (10 mm i.d.) operating between 280 and 380 °C under 3.0 MPa of system pressure. To activate the catalyst, a pre-reduction step was performed under a pure H_2_ flow at 400 °C and 3.0 MPa for a duration of 5 h. The feed was introduced at a weight hourly space velocity (WHSV) of 2 h^−1^, maintaining a constant H_2_ to n-C_16_ molar feed ratio of 10. The composition of the liquid products was determined by gas chromatography (GC) using a Chromatek-Crystal 5000 instrument (Chromatec, Yoshkar-Ola, Russia) equipped with a flame ionization detector and a 50 m long HP-1 capillary column.

## 4. Conclusions

In the present study, it is demonstrated that targeted variation in alkalinity (Na_2_O/SiO_2_ ratio) and hydrothermal synthesis time is an effective tool for controlling the properties of ZSM-48 zeolite. It was established that moderate alkalinity and reduced holding time (Na_2_O/SiO_2_ = 0.06, 48 h) allow for balancing the nucleation and growth rates, ensuring the formation of a phase-pure zeolite consisting of small aggregates of nanosized needle-like crystals with a high external surface area and a developed network of intercrystalline mesopores.

Conversely, high alkalinity and prolonged crystallization accelerate the process drastically, promoting aggregation into dense spherulites, a reduction in mesopore volume, and the appearance of impurity phases (EU-1, cristobalite, α-quartz). Furthermore, despite an increase in the total concentration of acid sites, a significant portion of strong Brønsted sites in the large spherulitic aggregates proves to be diffusionally inaccessible to bulky molecules, which is confirmed by the discrepancy between NH_3_-TPD and FTIR-pyridine spectroscopy data.

The bifunctional catalyst based on the nanosized Pt/Z48-06-2 with developed secondary porosity support provides minimal diffusional resistance and rapid desorption of primary products, achieving an isomer selectivity of 82% at a yield of 73%. In contrast, the use of the large-aggregate Pt/Z48-10-3 with diffusion-limited acidity leads to prolonged residence of molecules in the micropores, an increase in secondary hydrocracking, and a drop in maximum isomer yield to 46%.

Thus, the strategy for designing an effective isodewaxing catalyst based on nanosized ZSM-48, without the use of expensive secondary templates, requires strict kinetic control: limiting the hydrothermal synthesis time to prevent the coarsening of aggregates and to fix the short-crystalline, nanosized morphology, which ensures optimal mass transfer and high process selectivity.

## Figures and Tables

**Table 1:** Crystallinity, phase composition, and chemical composition of the crystallization products obtained from the parent gel with a composition of xNa_2_O·1.0SiO_2_·0.007Al_2_O_3_·0.02HMBr·25H_2_O at 160 °C.

Sample	x	SiO_2_/ Al_2_O_3_	Crystallization Time, h	Phase Composition	Crystallinity, %	I(002)/ I(110)
Z48-02-3	0.02	n.d.	72	Amorphous	0	n.d.
Z48-04-2	0.04	n.d.	48	ZSM-48	76	3.4
Z48-04-3	0.04	135	72	ZSM-48 + trace EU-1	87	2.9
Z48-06-2	0.06	119	48	ZSM-48	89	4.3
Z48-06-3	0.06	128	72	ZSM-48 + trace EU-1	91	4.3
Z48-08-2	0.08	114	48	ZSM-48	90	4.9
Z48-08-3	0.08	123	72	ZSM-48	91	5.6
Z48-10-2	0.10	113	48	ZSM-48	90	5.6
Z48-10-3	0.10	116	72	ZSM-48 + trace cristobalite	92	5.7
Z48-12-2	0.12	n.d.	48	ZSM-48 + α-quartz	92	5.5

SiO_2_/Al_2_O_3_—molar ratio based on XRF data; I(002)/I(110)—ratio of the integrated intensities of the reflections at 21.25 (002) and 7.61 (110); n.d.—not determined.

**Table 2:** Textural properties of ZSM-48 samples.

Sample	S_BET_, m^2^/g	S_EXT_, m^2^/g	V_micro_, cm^3^/g	V_meso_, cm^3^/g
Z48-06-2	176	58	0.06	0.09
Z48-08-2	209	52	0.06	0.06
Z48-10-2	191	48	0.08	0.06
Z48-04-3	185	51	0.07	0.09
Z48-06-3	165	49	0.07	0.07
Z48-08-3	168	51	0.05	0.03
Z48-10-3	179	42	0.07	0.06

S_BET_—specific surface according to BET. S_EXT_—external specific surface area. V_micro_—specific volume of micropores. V_meso_—specific volume of mesopores.

**Table 3:** Acidic properties of HZSM-48 zeolite samples according to NH3-TPD and FTIR-Pyridine (IR Py) data.

**Sample**	**Acidity by NH_3_ TPD, μmol/g**					
	**Weak ^a^**	**Moderate ^b^**		**Strong ^c^**		**Ʃ**
HZ48-06-2	88	39		68		195
HZ48-08-2	95	41		59		195
HZ48-10-2	110	41		72		223
HZ48-04-3	85	63		50		198
HZ48-06-3	83	62		56		201
HZ48-08-3	100	69		47		216
HZ48-10-3	102	43		84		229
	**Acidity by IR-Py, μmol/g**					
	**BAS**			**LAS**		
	**150 °C**	**250 °C**	**350 °C**	**150 °C**	**250 °C**	**350 °C**
HZ48-06-2	93	87	61	37	13	11
HZ48-08-2	82	79	56	46	17	13
HZ48-10-2	95	81	60	36	14	11
HZ48-04-3	78	67	54	49	22	16
HZ48-06-3	81	70	51	26	12	8
HZ48-08-3	59	54	37	11	4	3
HZ48-10-3	120	106	66	19	11	6

^a^ the amount of ammonia desorbed in the range of 100–250 °C; ^b^ the amount of ammonia desorbed in the range of 250–350 °C; ^c^ the amount of ammonia desorbed in the range of 350–600 °C; BAS—Brønsted acid sites; LAS—Lewis acid sites.

**Table 4:** Dispersion and particle size of Pt in Pt-containing samples obtained by H_2_ chemisorption.

Sample	Pt Content, %	H_2_ Uptake, μmol/g	Pt Dispersion, %	Pt Particle Size, nm
Pt/Z48-06-2	0.51	6.8	26	3.8
Pt/Z48-04-3	0.47	6.6	27	3.6
Pt/Z48-06-3	0.52	8.0	30	3.3
Pt/Z48-08-3	0.55	6.5	23	4.3
Pt/Z48-10-3	0.48	8.4	34	2.9

## Data Availability

The original contributions presented in the study are included in the article; further inquiries can be directed to the corresponding authors.

## Supplementary Materials

The following supporting information can be downloaded at: https://www.mdpi.com/article/10.3390/molecules31162900/s1, Figure S1. SEM images of ZSM-48 zeolite obtained at different crystallization times and Na_2_O/SiO_2_ molar ratios in the parent gel: (a) Na_2_O/SiO_2_ = 0.04, 12 h; (b) Na_2_O/SiO_2_ = 0.04, 24 h; (c) Na_2_O/SiO_2_ = 0.06, 12 h; (d) Na_2_O/SiO_2_ = 0.06, 24 h; (e) Na_2_O/SiO_2_ = 0.08, 12 h; (f) Na_2_O/SiO_2_ = 0.08, 24 h.
