# Supplementary material for: Alkali Content as a Tool for Tailoring ZSM-48 Physicochemical Properties: From Crystallization Kinetics to Catalytic Performance in n-Hexadecane Hydroisomerization
Source: Molecules. 2026 Aug 20;31(16):2900. doi: 10.3390/molecules31162900 (PMC13515814; doi:10.3390/molecules31162900)
Supplement: Supplementary file 1 [file molecules-31-02900-s001.zip › molecules-4458549-supplementary.pdf]

Alkali Content as a Tool for Tailoring ZSM-48 Physicochemical Properties: From Crystallization Kinetics to Catalytic Performance in n-Hexadecane Hydroisomerization

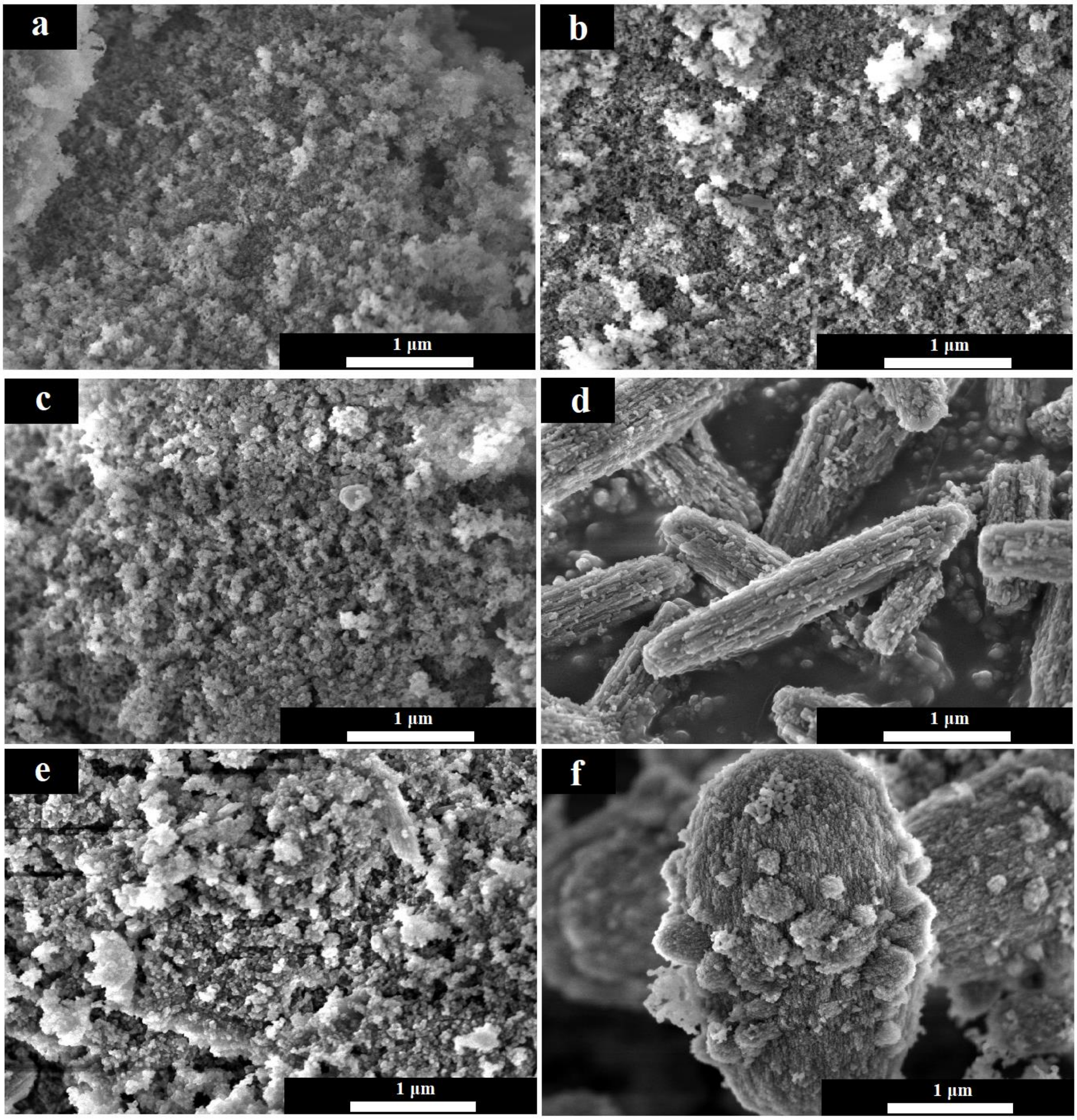

Figure S1. SEM images of ZSM-48 zeolite obtained at different crystallization times and Na_2O/SiO_2 molar ratios in the parent gel: (a) Na_2O/SiO_2 = 0.04, 12 h; (b) Na_2O/SiO_2 = 0.04, 24 h; (c) Na_2O/SiO_2 = 0.06, 12 h; (d) Na_2O/SiO_2 = 0.06, 24 h; (e) Na_2O/SiO_2 = 0.08, 12 h; (f) Na_2O/SiO_2 = 0.08, 24 h
